# Tranexamic acid is associated with selective increase in inflammatory markers following total knee arthroplasty (TKA): a pilot study

**DOI:** 10.1186/s13018-018-0855-5

**Published:** 2018-06-18

**Authors:** Andrea L. Grant, Hayley L. Letson, Jodie L. Morris, Peter McEwen, Kaushik Hazratwala, Matthew Wilkinson, Geoffrey P. Dobson

**Affiliations:** 1 The Orthopaedic Research Institute of Queensland (ORIQL), 7 Turner St, Pimlico, Townsville, Queensland 4812 Australia; 2 Heart, Trauma and Sepsis Research Laboratory, College of Medicine and Dentistry, James Cook University, 1 James Cook Drive, Townsville, Queensland 4811 Australia

**Keywords:** Tranexamic acid, Total knee arthroplasty, Coagulation, Inflammation, Orthopaedic surgery, Trauma

## Abstract

**Background:**

Tranexamic acid (TXA) is commonly used in orthopedic surgery to reduce excessive bleeding and transfusion requirements. Our aim was to examine if TXA was required in all osteoarthritis patients undergoing TKA surgery, and its possible effects on systemic inflammation and coagulation properties.

**Methods:**

Twenty-three patients (Oxford Score 22–29) were recruited consecutively; 12 patients received TXA before (IV, 1.2 g/90 kg) and immediately after surgery (intra-articular, 1.4 g/90 kg). Inflammatory mediators and ROTEM parameters were measured in blood at baseline, after the first bone-cut, immediately after surgery, and postoperative days 1 and 2.

**Results:**

After the bone cut and surgery, TXA significantly increased MCP-1, TNF-α, IL-1β and IL-6 levels compared to non-TXA patients, which was further amplified postoperatively. During surgery, TXA significantly prolonged EXTEM clot times, indicating a thrombin-slowing effect, despite little or no change in clot amplitude or fibrinogen. TXA was associated with three- to fivefold increases in FIBTEM maximum lysis (ML), a finding counter to TXA’s antifibrinolytic effect. Maximum lysis for extrinsic and intrinsic pathways was < 8%, indicating little or no hyperfibrinolysis. No significant differences were found in postoperative hemoglobin between the two groups.

**Conclusions:**

TXA was associated with increased systemic inflammation during surgery compared to non-TXA patients, with further amplification on postoperative days 1 and 2. On the basis of little or no change in viscoelastic clot strength, fibrinogen or clot lysis, there appeared to be no clinical justification for TXA in our group of patients. Larger prospective, randomized trials are required to investigate a possible proinflammatory effect in TKA patients.

## Background

A common perioperative complication during knee and hip surgery is excessive bleeding and the need for blood products [1, 2]. Serine protease inhibitor aprotonin was removed from world markets in 2007 and led to renewed interest in tranexamic acid (TXA) for reducing blood loss during major surgery [3, 4]. TXA is a synthetic lysine analog that reduces active bleeding by blocking the 5 lysine-binding sites on plasminogen, which prevents plasmin formation and decreases fibrinolysis [3, 5]. TXA has a plasma half-life of ~ 2 h, and its antifibrinolytic effects may last up to 7–8 h in the circulation, and ~ 17 h in most tissues [6].

In orthopaedic surgery, two injections of TXA are commonly used; one is given intravenously before the operation, and another in the knee joint on deflation of the tourniquet [7–9]. In a recent large retrospective cohort study, involving 872,416 patients, Poeran and colleagues concluded that TXA was effective in reducing the need for blood transfusions during total hip or knee arthroplasty [10]. However, despite the overwhelming evidence, the same authors cautioned that “we cannot provide support for the ubiquitous use of TXA in all patients requiring joint arthroplasty, as the differential impact on complications among patient subpopulations remains to be studied” [10]. A number of ongoing concerns include timing, dose, route of delivery (IV, oral, topical), and whether all patients should receive the drug [2, 8, 11]. Furthermore, there remains the risk of thromboembolic events [3, 12], and there is an increased awareness in the literature that TXA-specific lysine residues are not specific to reducing blood loss [13], but are involved in other metabolic and signalling events, protein-protein interactions and post-translational modifications [14]. In some cases, TXA can increase bleeding in brain independent of the tPA effect by binding to plasminogen in the presence of increased levels of urokinase plasminogen activator (uPA), which facilitates plasmin formation and the propensity to bleed [4, 15]. In 2017, we also showed that TXA administration in medium-risk cardiac surgery patients led to anomalous clot behaviour after a sternotomy, lower platelet numbers after surgery, and little or no difference in fibrinolysis compared to non-TXA patients [16].

A number of studies and many reviews have suggested that TXA may have anti-inflammatory properties via inhibition of plasmin-mediated activation of complement, monocytes, and neutrophils and may also improve platelet function [17, 18]. However, the evidence is weak, and since mediators of inflammation are associated with increased risk of thrombosis, and vice versa [13, 19], further studies are warranted. We hypothesized based on our previous cardiac surgery study [16] that TXA may have anomalous effects on coagulation properties during surgery, alter the patient’s inflammatory status and may not be required for all TKA patients. Thus, the aim of the present study was to examine the effects of TXA on coagulation and inflammation prior to, during, and following surgery, and assess if TXA was required in all patients undergoing elective TKA surgery.

## Methods

### Approvals

Informed consent was obtained prospectively from all participants, and the study was approved by the Institutional Human Research Ethics Committee (MHS20140812-03). The research undertaken strictly adhered to the Code of Ethics (Declaration of Helsinki) of the World Medical Association for trials involving humans. This study was an analytic, prospective, observational cohort (level II) investigation in which patient groups were separated non-randomly by treatment, with exposure occurring after the initiation of anesthesia.

### Subjects and procedures

Twenty-three patients (6 male, 17 female) undergoing TKA across three private practices were recruited to participate in the study (Table 1). The inclusion criteria were patients who were diagnosed with primary knee osteoarthritis (OA). The exclusion criteria were patients with (1) rheumatoid OA, (2) autoimmune disorders, (3) recent or recurrent infections with antibiotic treatment, or (4) contraindication for TXA use (thrombotic disorder and hematuria). Two of the three surgeons routinely use TXA perioperatively, and the remaining surgeon performed all surgeries for the non-TXA group.

**Table 1:** Demographics, comorbidities and pre-operative, perioperative and post-operative values (6 weeks) in non-TXA and TXA groups

	Non-TXA	TXA	*p* value
Age (years)	69 ± 1	65 ± 1	0.092
Weight (kg)	91 ± 5	90 ± 6	0.930
No. of patients	11	12	
Gender	M = 4	M = 2	
	F = 7	F = 10	
Osteoarthritis	11	12	
TXA administration:			
IV Infusion (mg/kg)	NA	13.5 ± 0.6	
IA Injection (mg/kg)	NA	15.5 ± 0.7	
Preoperative:			
Hemoglobin (HgB) g/L	136 ± 4	137 ± 4	0.980
Anesthetic:			
General only	0	1	
General + Spinal	11	11	
Perioperative:			
Tourniquet time (Min)	62 ± 11	27 ± 6	0.023*
Surgical time (Min)	107 ± 6	104 ± 4	0.566
Postoperative:			
HgB g/L day 1	121 ± 3^§^	122 ± 3^§^	0.787
HgB g/L day 2	111 ± 2^§^	121 ± 5^§^	0.093

Data represent mean ± SEM *p < 0.05 between non-TXA and TXA group;

^§^*p* < 0.05 compared to preoperative value

All patients with exception of one TXA patient received both spinal and general anesthesia (Table 1). The anesthetic procedure included intravenous administration midazolam (0.02 mg/kg) and propofol target-controlled infusion (2–5 mg/kg/h) with fentanyl (75–85 μg) as required. A tourniquet was inflated prior to the first midline skin incision and a medial parapatellar access was used to expose the joint capsule. After the bone cut, an intra-articular cocktail comprising Ropivacaine (400 mg), Ketorolac (30 mg), Adrenaline (1 mg), and Methylprednisolone (40 mg) in a total volume of 200 ml of saline was injected into the sub-synovial space, ligaments and muscles around the knee. All unilateral TKA surgery was assisted with Precision Computer Navigation (Stryker®). Implanted prosthesis (cruciate retaining) were cemented (PALACOS® R+G), Heraeus Medical, Germany). After implantation and tourniquet deflation, the capsule was closed.

### TXA administration

**Fig. 1:**
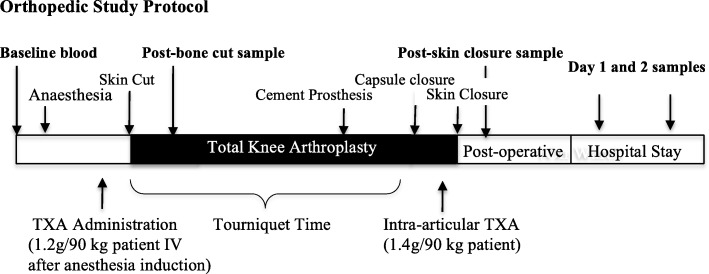
Summary of the study design and blood sampling times. Arterial blood was collected for rotational thromboelastometry (ROTEM) and measurement of plasma inflammatory cytokines and chemokines at (1) baseline before anesthesia, (2) 10 min after bone cuts, and (3) end of surgery (30 min after skin closure). Sampling was also conducted on days 1 and 2 postoperatively for routine hematology and inflammatory cytokine/chemokine assessment

Following induction of anesthesia and prior to skin incision, an intravenous bolus injection of TXA (1.2 g per 90-kg patient) was administered (Fig. 1, Table 1). After the operation and before skin closure, a second TXA bolus (15.5 g/kg body wt.) was injected in the intra-articular space in saline (i.e., 1.0 g TXA/10 ml saline).

### Clinical assessments

Patients were assessed preoperatively and followed up at 6 weeks postoperative for clinical assessments (goniometry for range of movement (ROM) as well as pain scores), and patient reported outcome measures, which included the Knee injury and Osteoarthritis Outcomes Score (KOOS), Oxford Knee Score (OKS), EuroQol (EQ-5D 3L) and Forgotten Joint Score (FJS) (Table 1). Practice nurses used the Angulus ROM iPhone app, which provides flexion and extension values by recording and measuring movement in both a horizontal and vertical plane.

### Blood sampling

Peripheral venous blood was collected from patients at three time points: (1) baseline, prior to anesthesia, (2) ~ 10 min after the first bone cut, and (3) ~ 30 min following skin closure. Blood was also collected from patients on days 1 and 2 postoperative. Whole blood (1.8 ml) was collected in 3.2% sodium-citrate vacutainers (BD Australia) for coagulation assessment, and 4 ml was collected into K_2_EDTA vacutainers (BD Australia) and centrifuged (1500 rpm, 15 min, 4 °C). Plasma was removed and snap-frozen in liquid nitrogen and stored at − 80 °C for cytokine measurements.

### Rotational thromboelastometry

**Fig. 2:**
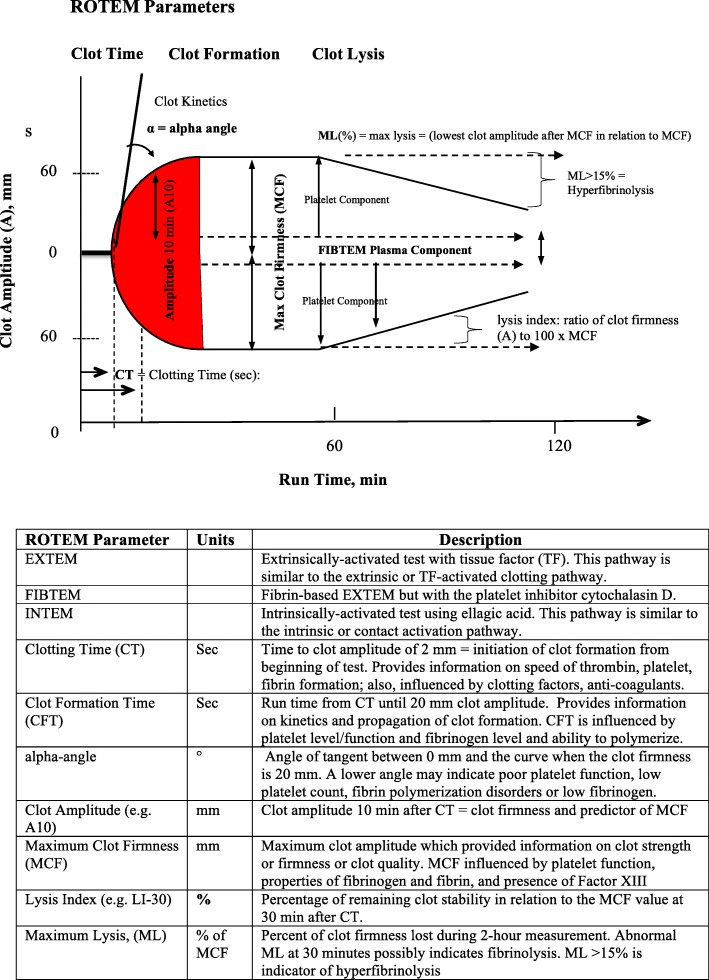
Schematic of a ROTEM Trace showing the key coagulation parameters measuring clot initiation, propagation and clot breakdown or lysis, and definitions of the major ROTEM parameters used in the study

Rotational thromboelastometry (ROTEM®, Tem International, Munich, Germany) was performed on ROTEM® delta according to the manufacturer’s instructions and described in Letson and Dobson [20] and Solomon and colleagues [21] (Fig. 2). Assays were run for 60 min. Hyperfibrinolysis was defined as a maximum lysis index greater than or equal to 15% [20, 22].

### Cytokine analysis

Milliplex® Human Cytokine/Chemokine Magnetic Bead Panel (Lot #: 2875005, Abacus ALS, Meadowbrook, Queensland) in combination with the Magpix® analyser (Luminex Corporation, Austin, Texas, USA) were used to measure plasma levels of monocyte chemotactic protein (MCP)-1, tumor necrosis factor alpha (TNF-α), interleukin (IL)-6, IL-8, IL-1β, IL-1 receptor antagonist (IL-1RA), IL-4 and IL-10 at baseline, 10 min after bone cut, in recovery, and on days 1 and 2 after surgery. Assays were carried out according to the manufacturer’s instructions with samples measured in duplicate. Detection ranges for all analytes were 3.2–10,000 pg/ml. Assay sensitivities (minimum detectable concentration, pg/ml), intra-assay precision (%CV) and inter-assay precision (*n* = 6 assays; %CV) for each analyte were MCP-1: 1.9, 1.5, 7.9; TNFα: 0.7, 2.6, 13.0; IL-6: 0.9, 2.0, 18.3; IL-8: 0.4, 1.9, 3.5; IL-1β: 0.8, 2.3, 6.7; IL-1RA: 8.3, 2.1, 10.7; IL-4: 4.5; 2.9, 14.7; IL-10: 1.1, 1.6, 16.8.

### Statistics

A priori power analysis to determine sample sizes was conducted using G-power3 program to minimize type 1 errors and was based on differences between coagulation parameters prior to and at surgery end [16]. A sample size of 10 patients in each group was sufficient for statistically valid comparisons to be made with respect to TXA vs non-TXA treatments with the power set at 0.8 and alpha level at 0.05. SPSS Statistics 24.0 was used for all data analysis (IBM, Armonk, NY). Data normality was assessed using Shapiro-Wilks test, with Levene’s test used to determine equality of variances. Independent samples *t* tests were used for between-groups comparison for normally distributed data. Within group differences were analysed with paired samples *t* tests. Non-normally distributed data was compared using a Mann-Whitney *U* test. MILLIPLEX Analyst 5.1 software (Luminex Corporation, Austin, Texas, USA), which analyses data with a 5 parametric logistic weighted curve fit, was used to determine cytokine concentrations. Area under the curve (AUC) was determined for changes in plasma cytokine levels across each of the five time points assessed. The mean AUC for each cytokine was compared for non-TXA and TXA patients using an independent-t test with Welch’s correction. All values are expressed as mean ± standard error of the mean (SEM) with significance set at *p* < 0.05.

## Results

### Perioperative characteristics

There were no significant preoperative differences in patient demographic or clinical parameters (Table 1). Tourniquet time was significantly less in TXA patients compared to non-TXA patients, with no significant differences in surgical times (Table 1). Lower tourniquet times may be due to differences in surgical procedures among surgeons; however, it is important to note that possible longer ischemic times in non-TXA patients may exacerbate postoperative inflammation; however, it was less than TXA-treated patients in our cohort (see below). No differences in preoperative knee biomechanic measures were observed, with the exception of significantly higher extension (5° vs. 2°) in the non-TXA group (Table 2). At 6 weeks postoperative, patients within each group demonstrated significant improvements in KOOS measures compared to baseline, with no significant differences between scores for non-TXA and TXA patients (Table 2).

**Table 2:** Preoperative and postoperative (6 weeks) range of motion (ROM) and patientreported outcome measures (PROM) in non-TXA and TXA groups

	Non-TXA	TXA	*p* value
Preoperative:			
ROM			
Flexion (°)	115 ± 5	121 ± 5	0.411
Extension (°)	5 ± 1	2 ± 1	0.046*
KOOS Total	46 ± 6	41 ± 4	0.470
KOOS Pain	10 ± 2	8 ± 1	0.417
KOOS Function	32 ± 4	29 ± 3	0.457
KOOS Movement	4 ± 0.7	4 ± 0.4	1.000
OKS	22 ± 3	29 ± 3	0.457
EQ5D 3L VAS	64 ± 7	67 ± 4	0.760
Postoperative 6 weeks:			
ROM			
Flexion (°)	104 ± 5	110 ± 4^§^	0.297
Extension (°)	7 ± 1	4 ± 1	0.134
KOOS Total	26 ± 4^§^	17 ± 3^§^	0.096
KOOS Pain	5 ± 1^§^	4 ± 1^§^	0.304
KOOS Function	18 ± 3^§^	11 ± 2^§^	0.098
KOOS Movement	3 ± 0.5	2 ± 0.3^§^	0.063
OKS	27 ± 2	32 ± 2	0.097
EQ5D 3L VAS	67 ± 7	75 ± 4	0.339
FJS	62 ± 4	52 ± 6	0.170

Data represents mean ± SEM

*KOOS* The Knee Injury and Osteoarthritis Outcomes Score, *OKS* Oxford Knee Score, *EQ5D (3L)* EuroQol 5-Dimension 3-Level Assessment, *FJS* Forgotten Joint Score. *p* < 0.05 between non-TXA and TXA group; ^§^*p* < 0.05 compared with preoperative value

### Inflammatory status before, during and following surgery

**Fig. 3:**
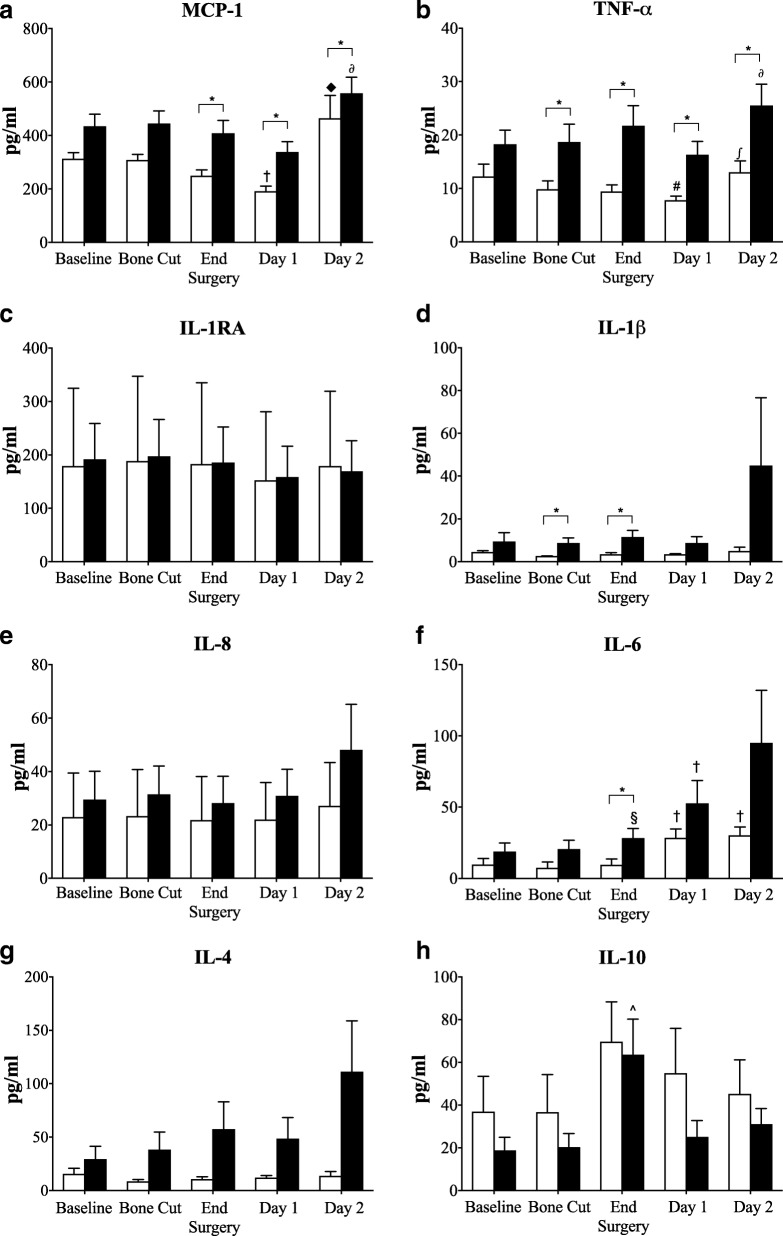
Plasma levels of inflammatory cytokine/chemokines **a** MCP-1, **b** TNF-α, **c** IL-1RA, **d** IL-1β, **e** IL-8, **f** IL-6, **g** IL-4, and **h** IL-10 at baseline, after bone cuts, surgery end, day 1 and day 2. White square: non-TXA group; black square: TXA group. Data is expressed as mean ± S.E.M. *p < 0.05 compared with corresponding non-TXA patients, ^◆^*p* < 0.05 compared to baseline, bone cut, end surgery and day 1, ^†^*p* < 0.05 compared to baseline, bone cut and end surgery; ^#^*p* < 0.05 compared to baseline; ^∫^*p* < 0.05 compared to end surgery and day 1; ^§^*p* < 0.05 compared to bone cut; ^^^*p* < 0.05 compared to baseline and bone cut

**Fig. 4:**
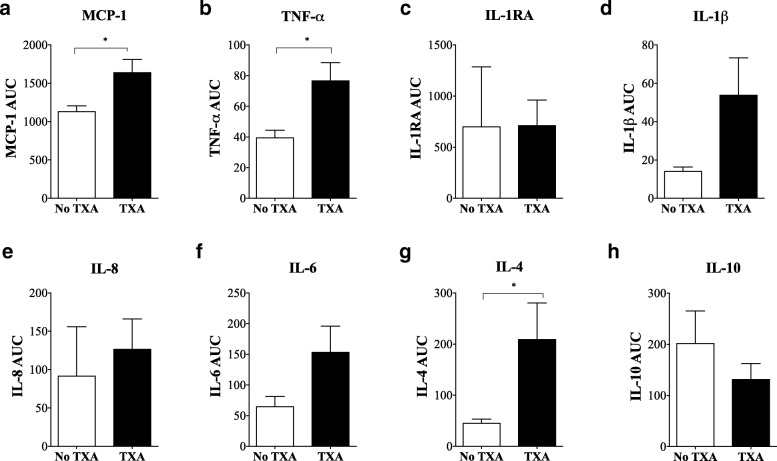
Area under the curve (AUC) for **a** MCP-1, **b** TNF-α, **c** IL-1RA, **d** IL-1β, **e** IL-8, **f** IL-6, **g** IL-4, and **h** IL-10 based on plasma cytokine levels kinetics from baseline to day 2 postoperative. White square: non-TXA group; black square: TXA group. Data is expressed as mean ± S.E.M. *p < 0.05 compared with corresponding non-TXA patients

There were no significant differences in baseline plasma inflammatory mediators between non-TXA and TXA patients (Fig. 3). At surgery end and postoperative days 1 and 2, patients that received TXA had significantly higher plasma levels of MCP-1 compared to non-TXA patients (Fig. 3). Area-under-the-curve (AUC) analysis over 3 days supported this finding (*p* = 0.013) (Fig. 4). TNF-α was also significantly higher in TXA patients at each of the time points assessed (Fig. 3), and supported by AUC analysis (*p* = 0.010). IL-6 was significantly higher immediately after surgery and 1.8-times higher than non-TXA group on day 2 postoperative, but did not reach significance. Similarly, IL-8, a chemokine attractant for neutrophils and lymphocytes, and inducible by TNF-α and IL-1β [23], was 1.8 times higher on day 2 (*p* = 0.085) in TXA versus non-TXA patients. Levels of IL-1β, an inflammation amplifier, were also elevated in plasma of TXA patients after the first bone cut and at surgery end (Fig. 3). However, despite a ninefold increase in IL-1β in TXA patients compared to the non-TXA group at day 2 postoperative (Fig. 3) and a threefold higher AUC value (Fig. 4; *p* = 0.064), these differences were not significant. Plasma levels of IL-1RA remained unchanged throughout surgery through to postoperative day 2 (Fig. 3). Plasma IL-4 levels were higher in TXA patients compared to non-TXA patients after the first bone cut and at surgery end, with levels continuing to increase on day 2. The AUC for IL-4 was significantly higher (8 times) for TXA than non-TXA patients (*p* = 0.042, Fig. 4). After surgery, the anti-inflammatory cytokine IL-10 peaked in both patient groups, and then decreased on days 1 and 2, with a trend toward higher values of IL-10 in plasma from non-TXA compared to TXA patients (1.45 to 2.2 times higher) (Fig. 3).

### Coagulation parameters

#### EXTEM

**Fig. 5:**
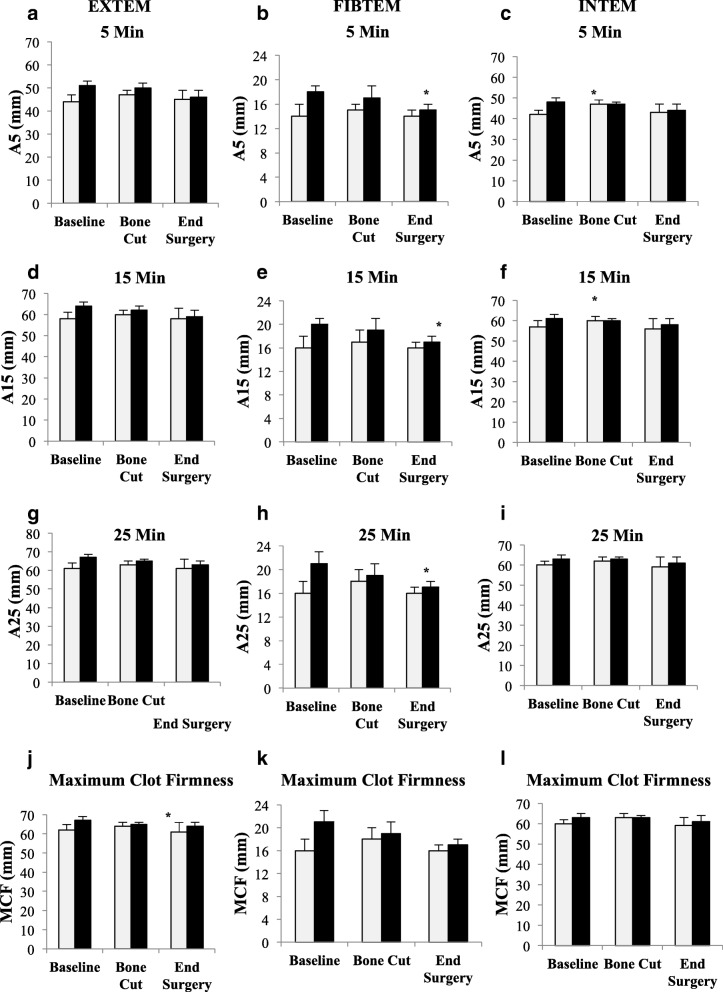
EXTEM, FIBTEM and INTEM clot amplitudes at 5 (A5, mm), 15 (A15, mm), 25 min (A25, mm) and Maximum Clot Firmness (MCF, mm) at baseline, after bone cuts, surgery end, day 1 and day 2. White square: non-TXA group; black square TXA group. Data expressed as mean ± S.E.M. *p < 0.05 compared to baseline

In non-TXA patients after the first bone cut and at surgery end, CT and α-angles were similar to baseline (Table 3), although CFTs fell by ~ 20% (96 to 76 s) suggesting a slowing of clot elongation (Fig. 2). Clot amplitudes underwent little or no change in non-TXA patients (Fig. 5), as did clot lysis (LI30, LI45 or ML) (Table 3). In direct contrast, TXA led to significant increases in CT after the bone cut and surgery compared to non-TXA patients, with no additional coagulation changes observed (Table 3, Fig. 5).

**Table 3:** Clot kinetics and lysis parameters for TXA and non-TXA patients at baseline, after bone cut, and end of surgery as measured on EXTEM, FIBTEM and INTEM tests

Test	Group	Time	CT (s)	CFT (s)	Alpha Angle (°)	LI30 (%)	LI45 (%)	ML (%)
EXTEM	Non-TXA	Baseline	59 ± 3	96 ± 2	72 ± 3	100 ± 0	98.1 ± 0.4	3.1 ± 1.0
		Bone Cut	53 ± 1	76 ± 7	76 ± 2^*^	100 ± 0	97.4 ± 0.5	6.3 ± 0.8*
		Surgery End	51 ± 3	75 ± 7	73 ± 3	100 ± 0	98.7 ± 0.3^¥^	3.9 ± 0.6^¥^
	TXA	Baseline	64 ± 4	70 ± 7	76 ± 1	98.8 ± 1.2	95.6 ± 1.9	7.9 ± 1.9
		Bone Cut	59 ± 5	67 ± 6	78 ± 1	99.8 ± 0.3	97.3 ± 0.6	6.5 ± 1.0
		Surgery End	65 ± 3^¶^	89 ± 14	73 ± 2	100 ± 0	98.3 ± .4	4.4 ± 0.8
FIBTEM	Non-TXA	Baseline	59 ± 6	884 ± 476	76 ± 1	99.1 ± 0.9	99.3 ± 0.7	1.4 ± 1.3
		Bone Cut	53 ± 3	797 ± 604	73 ± 2	100 ± 0	99.9 ± .10	0.5 ± 0.3
		Surgery End	49 ± 2	1483 ± 576	67 ± 7	99.9 ± 0.1	100 ± 0	0.4 ± 0.2
	TXA	Baseline	66 ± 5	1020 ± 480	74 ± 2	100 ± 0	99.5 ± 0.23	1.1 ± 0.4
		Bone Cut	54 ± 4^*^	403 ± 300	76 ± 1^*^	98.8 ± 0.7	98.3 ± 1.1	2.8 ± 1.3
		Surgery End	68 ± 5^¶^	480 ± 301	70 ± 2	99.9 ± 0.1	99.8 ± 0.2	1.1 ± 0.4
INTEM	Non-TXA	Baseline	204 ± 15	104 ± 16	71 ± 2	99.7 ± 0.3	97.7 ± 0.7	4.6 ± 1.0
		Bone Cut	164 ± 10*	71 ± 8*	76 ± 2*	99.7 ± 0.2	96.6 ± 0.5	6.3 ± 0.6
		Surgery End	162 ± 13*	69 ± 10*	73 ± 2	100 ± 0	98.3 ± 0.4	4.1 ± 0.8
	TXA	Baseline	170 ± 11	69 ± 8^¶^	76 ± 2^¶^	99.7 ± 0.1	95.9 ± 0.5	6.8 ± 0.8
		Bone Cut	149 ± 8*	65 ± 4	77 ± 1	99.8 ± 0.1	97 ± 0.6^*^	5.8 ± 0.7
		Surgery End	154 ± 12	101 ± 31	73 ± 4	100 ± 0*	97.9 ± 0.4^§^	4.8 ± 0.7^§^

Data represent mean ± SEM. *CT* clot time, *CFT* clot formation time, *LI* lysis index, *ML* maximum lysis. *n* = 12 for TXA group; *n* = 11 for non-TXA group. *p < 0.05 compared with baseline; ^¥^*p* < 0.05 compared with Bone Cut; ^§^*p* < 0.05 compared with baseline and bone cut; ^¶^*p* < 0.05 compared non-TXA group

#### FIBTEM

Following surgery, TXA led to a significant increase (1.4 times) in FIBTEM CT compared to non-TXA patients (*p* = 0.004) (Table 3). There were no differences in FIBTEM amplitudes at baseline, after bone cut and at surgery end (Fig. 5), indicating that fibrinogen concentration remained unchanged during TKA surgery. TXA also increased maximum lysis following the first bone cut (0.5% to 2.8%, *p* = 0.389) and at surgery end (0.4 to 1.1%. *p* = 0.140); however, these differences were not significant (Table 3).

#### INTEM

In both TXA and non-TXA patients, CT values were lower than their respective baseline values after the first bone cut or at surgery end, with no statistical difference between groups (Table 3). There were also no differences in clot amplitude at any time point (Fig. 5). Clot lysis was also comparable between the groups and ranged from 4.1 to 6.8%, indicating little or no hyperfibrinolysis.

## Discussion

Antifibrinolytics are widely used in orthopaedic surgery to reduce excessive bleeding and minimize transfusion requirements and re-exploration [5]. In our pilot study in OA patients undergoing TKA, we report:

- Elevated baseline plasma levels of MCP-1 and TNF-α relative to healthy, aged-matched human values, indicating the presence of low-grade systemic inflammation prior to surgery.
- After the first bone cut and surgery end, MCP-1, TNF-α, IL-1β and IL-6 (after surgery) were significantly increased in TXA compared to non-TXA patients, with differences further amplified at postoperative days 1 and 2. TXA appeared to exacerbate the surgical stress inflammatory response.
- EXTEM CT was prolonged in TXA patients after the first bone cut and at surgery end, indicating a thrombin-slowing effect on clot initiation, despite little or no change in clot amplitude or fibrinogen levels.
- In TXA patients there was a tendency for increased FIBTEM maximum lysis during surgery, a finding that is counter to TXA’s antifibrinolytic effect.
- Maximum lysis in EXTEM and INTEM was < 5% and < 7%, respectively during surgery, indicating little or no hyperfibrinolysis, and supported by similar falls in hemoglobin levels (11–19%) on days 1 and 2 postoperative relative to baseline. These data question the need for TXA in this surgical setting.

### Low-grade inflammation in OA patients

In chronic OA patients, joint inflammation appears to be expressed systemically as a low-grade inflammatory state [24–29]. We found that baseline levels of plasma MCP-1 (CCL2) were up to four times higher than in aged-matched healthy individuals (95–168 pg/ml) [30, 31], and TNF-α levels almost three times higher than in normal humans (~ 5 pg/ml) [31, 32] (Fig. 3). MCP-1 is a chemokine that regulates recruitment of immune cell traffic from the circulation to sites of inflammation in OA patients [33, 34], and has been implicated in articular cartilage degradation and pain [27, 35]. TNF-α is another potent inflammatory mediator involved in OA progression [36, 37], contributing to cartilage loss through its suppression of collagen and proteoglycan synthesis [24, 36, 38]. Notwithstanding the difficulty of finding aged-matched healthy human data, our data suggest the OA patients in the current study presented with a low-grade systemic inflammation.

### TXA exacerbates inflammation in response to surgical stress

We report significant increases in plasma IL-1β and TNF-α in TXA patients after the first bone cut and at surgery end compared to patients that did not receive TXA (Fig. 3). At the end of surgery, TNF-α and IL-1β continued to increase in TXA patients and were accompanied by significantly higher IL-6 and MCP-1 compared to non-TXA patients (Fig. 3). Although the cytokine increases were small, they indicate a heightened inflammatory state in the TXA patients, and heightened surgical stress response [39–41]. During this early period in knee or hip surgery, Hall and colleagues have confirmed activation of the surgical stress response involving the hypothalamic–pituitary–adrenal **(**HPA) axis, with concomitant increases in plasma cortisol and catecholamines [42]. In TKA, the stress response is most likely activated from multiple neural, hormonal and metabolic inputs including danger signals (e.g. alarmins) from, soft tissue and bone resection, and firing of afferent nerves, that are detected by resident and circulating immune cells, and the brain respectively [40].

In addition to TXA exacerbating the inflammatory response *during* surgery, another key finding was the apparent amplifying effect of TXA on inflammatory cytokine levels over the first two postoperative days (Fig. 3). We found increased concentrations of plasma MCP-1, TNF-α, IL-1β, IL-6, IL-8, and IL-4 and decreased IL-10 levels in patients that received TXA compared to those that did not. AUC analysis from baseline to postoperative day 2 showed significantly higher levels of MCP-1, TNF-α and IL-4 in plasma of TXA than in non-TXA patients (Fig. 4). The differences in IL-4 are of particular interest since it is generally regarded as an anti-inflammatory cytokine, similar to IL-10 and IL-13 [43]. In this role, IL-4 is known to inhibit TNF-α production and IL-1β synthesis and to increase IL-1RA [43, 44]. However, the opposite occurred in TXA patients in our study. At postoperative day 2, plasma TNF-α levels were twofold higher, and IL-1β was fivefold higher compared to non-TXA patients, with no change in IL-1RA (Fig. 2).

Recently, Major and colleagues also reported that IL-4 was not purely an anti-inflammatory cytokine, but could prime macrophages, increase TNF-α and increase inflammation [44]. IL-4, in combination with GM-CSF, can further promote inflammation by increasing differentiation of monocytes into dendritic cells [44]. Bellini and colleagues also showed that IL-4 can stimulate a unique circulating leukocyte subpopulation (0.1–0.5%) of bone marrow-derived stem cells known as fibrocytes that leave the blood and enter the site of healing and differentiate into fibroblasts/myofibroblasts with increased production of cell matrix components, growth factors, and inflammatory cytokines [45–47]. Therefore, in the current study, it is possible that IL-4 contributes to a heightened systemic inflammatory response observed in TXA patients (Figs 3, 4).

### TXA prolonged clot times during surgery and had no effect on clot lysis

In our study, baseline ROTEM clotting parameters for OA patients were similar to normal healthy individuals [22, 31, 32, 48]. In contrast to a low-grade inflammatory state at baseline in our OA groups, it appears that there were no apparent coagulation defects. However, after the first bone cut and surgery end, the non-TXA patients had decreased EXTEM, FIBTEM and INTEM clot times (9 to 21% falls relative to baseline) (Table 3), indicating increased thrombin availability. This was further supported by 22 and 34% decreases in EXTEM CFT and INTEM CFT, respectively, with little or no change in α-angles (Table 3). The shift in CT and CFT was associated with no effect on clot amplitude or strength (Fig. 3) but a twofold increase (3.1 to 6.3%) in maximum lysis in EXTEM (*p* = 0.021) and 1.4 times (4.6 to 6.3%) in INTEM after the bone cut (Table 3). Notably, the increases in fibrinolysis in non-TXA patients were within the range of normal values, with 15% often used as a guide to indicate hyperfibrinolysis [20, 22]. Thus, in non-TXA patients, surgical stress appeared to decrease clot times and thrombin availability without changes in other ROTEM parameters.

However, in TXA patients, EXTEM CT at surgery end was significantly higher (1.3-fold) than non-TXA patients, indicating that TXA during surgical stress has a thrombin-slowing effect (Table 3). We reported a similar finding in cardiac surgery after a sternotomy with TXA having a twofold increase in CT (all tests) [16]. In that study, we speculated that TXA may (1) reduce the rate of prothrombin-thrombin conversion, or (2) inhibit one or more of the polypeptide cleavage reactions, and thereby slow the fibrinogen to fibrin conversion [16]. Interestingly, since TXA is a lysine analogue, reducing the prothrombin-thrombin conversion is possible since the kringle-2 domain of the prothrombin complex is rich in lysine residues, and TXA may partially block these sites thus reducing thrombin production. In contrast to our cardiac surgery study, both TXA and non-TXA patients had significantly decreased INTEM CT during surgery in the current study (Table 3), highlighting differences in TXA with clotting factors or pathway selection. Importantly, and in agreement with our previous study, we found no difference in EXTEM or INTEM clot lysis in TXA and non-TXA patients. This finding suggests that perhaps the beneficial effect of TXA published in a large number of randomized controlled trials involving nearly 1 million patients [10] might not be reflected by the absence of evidence of hyperfibrinolysis with ROTEM (and TEG). In addition, we found no difference in hemoglobin levels between the groups postoperatively (Table 1), suggesting blood loss was similar for both TXA and non-TXA patients after TKA surgery.

### TXA paradoxically increases FIBTEM maximum lysis after bone cut and surgery

Another interesting trend observed in the present study was that TXA increased maximum lysis in FIBTEM after the bone cut (5-fold higher) and the end of surgery (~ 3-fold higher) compared to non-TXA patients (Table 3). The FIBTEM test is EXTEM with platelet inhibition (see Fig. 2), and these paradoxical results suggest that TXA weakens, not strengthens, the fibrin network in the absence of platelet contribution. This pro-fibrinolytic effect of TXA was not due to falling levels of fibrinogen because there was no change in FIBTEM amplitudes (Fig. 5). Currently, we do not know the underlying mechanisms for this effect of TXA on maximum lysis. Platelets normally support the formation of a dense, stable fibrin network from αIIbβ3 integrin interactions and the fibrin network [49]. In the absence of platelets, it appears that lysine residues play a role in securing fibrin density and stability in the FIBTEM clot, which is decreased in the presence of TXA. While this observation may be clinically silent under normal hematological conditions, it has the potential to become a significant problem in major surgery or various trauma states, where platelet numbers may decrease or platelet activation is impaired numbers or function.

### Potential clinical significance: a call for precision-based medicine

An important finding in the present study was that there appeared to be no clinical advantage of using TXA in our patient group undergoing elective TKA. Without evidence of hyperfibrinolysis (Table 3), there is no clinical justification for TXA use because there is no excessive bleeding [4]. In addition, TXA administration appeared to have a potentially untoward pro-inflammatory effect during and after surgery, which may be linked to a more pronounced TXA-induced stress response to the trauma of surgery. This may be clinically significant since Galvez and colleagues recently demonstrated that patients with OA already have a diminished ability to tolerate surgical stress [29], which the authors associated with a pre-existing low-grade chronic systemic inflammation [40, 42]. Our study further underscores a number of outstanding questions on TXA use in major surgery: (1) Would a single dose administration of TXA have less effect to increase inflammation and stress response to surgery? (2) What laboratory tests should be used to drive TXA use in elective or emergency surgery? and (3) What is the scientific basis for using TXA in orthopaedic surgery? In our view, *TXA should not be viewed as a one-size-fits-all approach to elective surgery*; rather it should be incorporated into a more precision-based set of guidelines [4, 50, 51]. The potential harmful effects of TXA on promoting inflammation warrant further investigation.

### Limitations of the study

A major limitation of our pilot study was its lack of randomization, blinding and small patient numbers. Our postoperative period was also limited and requires extension beyond day 2 when joint swelling is at a maximum, and 10–14 days when adhesions begin to form. Given surgically induced inflammation is also linked to postoperative pain and fragmented sleep patterns following TKA [42, 52, 53], these additional metrics should be included in future studies. Another limitation was that we did not measure plasma stress hormones, which may be higher in patients with higher inflammatory status during and following surgical stress [39–42]. We also do not know the effect of the cocktail components that were injected around the knee after the bone cut on TXA’s effect to change some ROTEM parameters and/or inflammatory markers. In vitro studies are also required to examine TXA’s effect on fibrinogen with and without platelets, and role of lysine residues using rapid-kinetic monitoring, X-ray crystallography, nuclear magnetic resonance and electron microscopy techniques. Notwithstanding these limitations, our study provides a springboard for a larger prospective, randomized trial to further elucidate the effects of TXA on inflammation and the surgical stress response to TKA, the outcomes of which may have implications for other pediatric and adult elective and emergency surgeries.

## Conclusions

In moderate-to-severe OA patients, TXA led to prolongation of EXTEM CT after the first bone cut and end of surgery compared to non-TXA patients, despite little or no change in clot strength or fibrinogen levels. Maximum lysis in EXTEM and INTEM was < 10% in both TXA and non-TXA patients, indicating little or no hyperfibrinolysis and thus questioning the need for TXA in our patient group. TXA was also associated with increased systemic inflammation, with rising plasma levels of proinflammatory cytokines in the first 2 days after TKA surgery.

## Abbreviations

IL-4: Interleukin 4
A10: Clot amplitude after 10 min
IL-1RA: Interleukin-1 receptor antagonist
CFT: Clot formation time
CT: Clot time
EXTEM: Extrinsically activated test with tissue factor
FIBTEM: Fibrin-based EXTEM with platelet inhibition
IL-1: Interleukin 1 beta
IL-10: Interleukin 10
IL-6: Interleukin 6
IL-8: Interleukin 8
INTEM: Intrinsically activated test using ellagic acid
LI: Lysis index
MCF: Maximum Clot Firmness
MCP-1: Monocyte chemoattractant protein-1
ML: Maximum lysis
ROTEM: Rotational Thromboelastometry
TKA: Total knee arthroplasty
TNF-α: Tumor necrosis factor alpha
tPA: Tissue plasminogen activator
TXA: Tranexamic acid
uPA: Urokinase plasminogen activator
